# Differential diagnosis of Guillain-Barré syndrome: steroid-responsive radiculopathy in Evans syndrome

**DOI:** 10.1186/s42466-024-00344-1

**Published:** 2024-09-20

**Authors:** Thomas Schulten, Ansgar Meyer, Utz Krug, Helmar C. Lehmann

**Affiliations:** 1 Department of Neurology, Klinikum Leverkusen, Am Gesundheitspark 11, 51375 Leverkusen, Germany; 2 Med360° Department of Radiology, Leverkusen, Germany; 3 Department of Internal Medicine, Klinikum Leverkusen, Leverkusen, Germany; 4 Medical Faculty, University Hospital of Cologne, Köln, Germany

**Keywords:** Guillain-Barré syndrome, Nerve conduction, Cerebrospinal fluid, Immune thrombocytopenia, Autoimmune hemolytic anaemia

## Abstract

Guillain-Barré syndrome is the most common acute inflammatory demyelinating peripheral nerve condition. Occasionally, other autoimmune conditions can mimic Guillain-Barré syndrome but may require different diagnostic workup and treatment. We report here two patients with Evans syndrome, a rare hematological autoimmune condition who developed a subacute inflammatory radiculopathy. Similarities and distinguishing clinical and diagnostic features are discussed.

## Background

Evans syndrome is a rare autoimmune condition clinically characterized by concomitant or sequential occurrence of immune thrombocytopenia (ITP) and autoimmune hemolytic anemia (AIHA) [1]. It is often associated with other autoimmune conditions (e.g. systemic lupus erythematosus), infections or hematological malignancies [1, 2]. Rarely, acute [3–5], and chronic [6, 7] inflammatory demyelinating peripheral neuropathies were reported in patients with Evans syndrome. We describe here the two patients with Evans syndrome who developed a subacute inflammatory radiculopathy.

## Methods

Among four patients with Evans syndrome, who were treated in the last ten years in our hospital, we reviewed two cases of patients with Evans syndrome and inflammatory radiculopathy retrospectively. The patients or their relatives gave written informed consent.

## Results

### Case 1

A male patient was diagnosed of primary AIHA of mixed type at the age of 24 years. Diagnosis was made on typical clinical features i.e. splenomegaly, positive Coombs test and spherocytes. After a phase of remission, two years later, ITP occurred, which subsequently lead to the diagnosis of Evans syndrome. In addition, the patient developed an autoimmune neutropenia one month after the diagnosis of ITP with high-titric antibodies against FcRIIIb. After refractoriness against glucocorticoids, the thrombopoietin-agonist eltrombopag, and the anti-CD20 antibody rituximab, the patient underwent splenectomy. After 14 months of remission, ITP relapsed at the age of 29 years. This relapse was treated with dexamethasone 40 mg orally for 4 days with prompt thrombocyte count response.

**Fig. 1:**
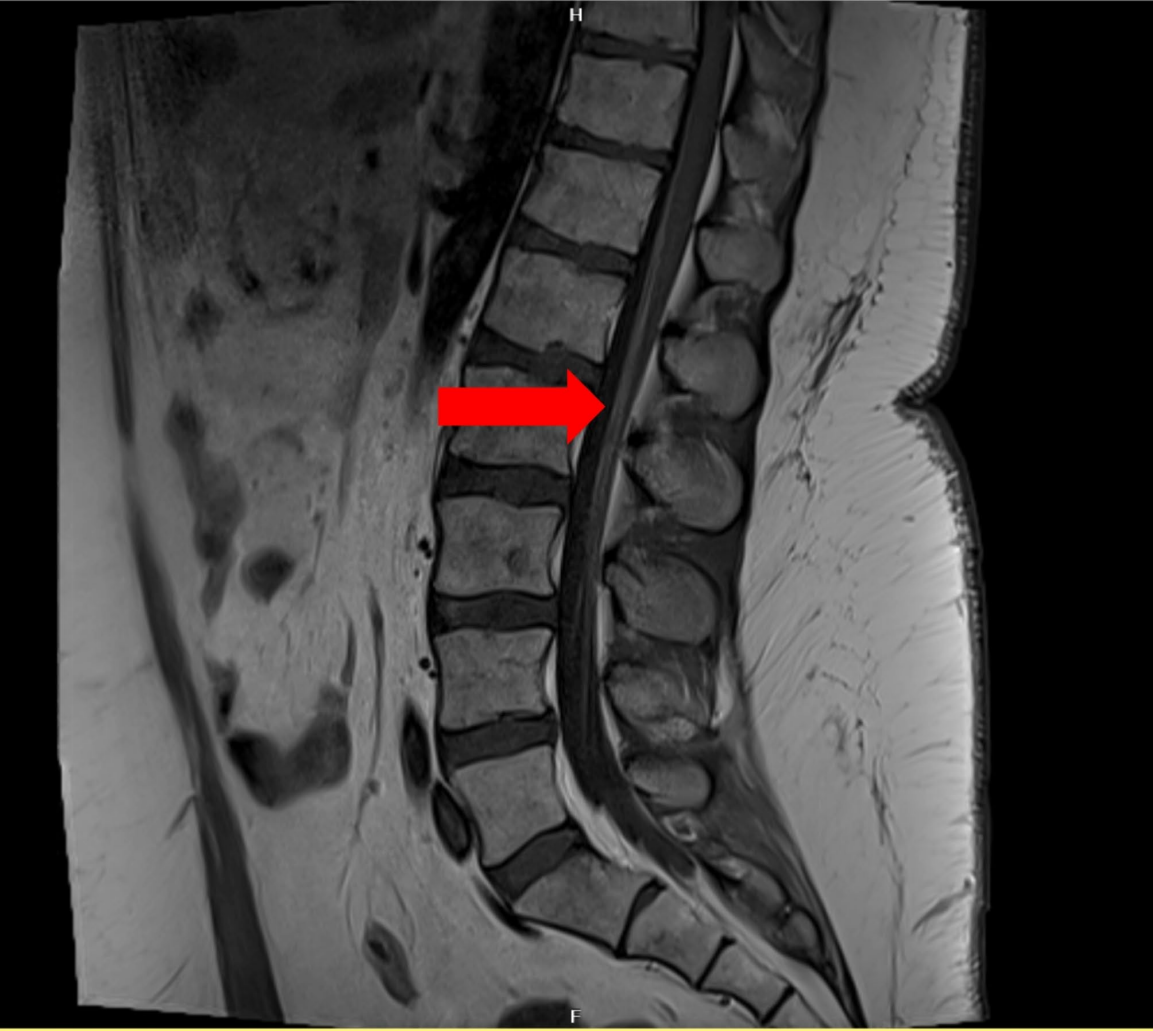
T1w-MRI with gadolinium of the lumbar spine steroid-responsive radiculopathy in Evans Syndrome (Arrow: gadolinium enhancement)

Fifteen days after commencement of treatment, the patient presented to our department with progressive walking difficulties and sensory disturbances in both legs that developed over 7 days. Neurological examination revealed a symmetrically reduced paralysis of the proximal and distal lower limbs. Sense of vibration, fine touch, and proprioception were normal. Gait was ataxic. The deep tendon reflexes were initially brisk, except for the Achilles tendon reflex which were weak. Babinski sign was negative. Cerebrospinal fluid (CSF) examination revealed initially pleocytosis of 50 cells per µl and normal protein concentration. Nerve conduction studies (NCS) revealed reduction of compound muscle action potentials (CMAPs) of the peroneal and tibial nerves. The patient underwent a thorough diagnostic workup including testing for other autoimmune or infectious causes of peripheral neuropathy. Central nervous system pathology was also ruled out by MRI. Lumbosacral radiculitis was suspected, and the patient was treated eventually 10 days after symptom onset with methylprednisolone intravenously which improved the neurological deficit. After discontinuation, the patient relapsed six weeks later with weak muscle reflexes. Plasmapheresis (6 courses) was initiated which lead to sustained remission. Another MRI of the lumbar spine with an injection of gadolinium, five months later, still demonstrated a gadolinium enhancement of the medullary conus and the cauda equina (Fig. 1).

### Case 2

This patient was diagnosed with Evans syndrome due to an ITP and concomitant AIHA at the age of 15. The patient was in continuous remission after splenectomy at the age of 19. When he was 24-years old, he presented in our department with tingling of the legs and paresis of the foot muscles. Symptoms had started five days earlier. On neurological examination the patient showed weak deep tendon reflexes, the Achilles reflexes were absent. Babinski sign was negative. CSF analysis unveiled a mild pleocytosis (15 cells per µl) but normal levels of protein, glucose and lactate. NCS disclosed a reduction of CMAPs of the peroneal nerves. MRI of the lumbar spine was unremarkable. Our patient was tested negative for infectious causes of radiculitis including HIV and SARS-CoV-2.

The patient was initially treated with antiinfective treatment until Lyme disease and herpes radiculitis were excluded. Subsequently, three days after presentation, the patient was treated with high-dose methylprednisolone intravenously (1000 mg) for five days. The patient showed a gradual improvement of the neurological symptoms and was discharged. Relapses were not observed.

## Discussion

We report here two patients that presented with a polyradiculitis resembling GBS. Compatible with GBS was particularly the acute onset in the two cases. However, in contrast to GBS, the two patients did not report any antecedent infections, which occurs in 76% of GBS cases [8, 9]. Other clinical features our patients displayed and which are less common in GBS were hyperreflexia (case#1), which only occurs in 2% of cases and rare variants [8, 10] and pleocytosis in the CSF, which is observed in only 19% of GBS cases, according to the IGOS cohort [8]. NCS showed in both cases a primarily axonal-radicular damage which is different to changes typical seen in GBS. Notably, the two patients did respond well to corticosteroids, although patient#1 relapsed after discontinuation. This steroid responsiveness stands in stark contrast to clinical and trial experience in GBS which usually is considered insensitive to steroids. The relapsing course of patient #1 may also be compatible with acute onset chronic inflammatory demyelinating polyneuropathy (CIDP), but the NCS displayed an axonal pattern, which is uncommon in CIDP.

## Conclusions

Lumbosacral radiculitis is a rare complication that may occur in patients with Evans syndrome. It shares clinical features of classical GBS, but to our experience responds well to steroids, which therefore can be considered as treatment alternatively to IVIg in this rare constellation.

## Data Availability

Available upon reasonable request.

## Abbreviations

AIHA: autoimmune hemolytic anemia
CIDP: chronic inflammatory demyelinating polyneuropathy
CSF: cerebrospinal fluid
CMAP: compound muscle action potential
GBS: Guillain-Barré syndrome
HIV: human immunodeficiency virus
IGOS: international GBS outcome study
ITP: immune thrombocytopenia
MRI: magnetic resonance imaging
NCS: nerve conduction studies
